# Removing Fractured Endodontic Files with a Tube Technique—The Strength of the Glued Joint: Tube-Endodontic File Setup

**DOI:** 10.3390/ma16114100

**Published:** 2023-05-31

**Authors:** Katarzyna Olczak, Jacek Grabarczyk, Witold Szymański

**Affiliations:** 1 Department of Endodontics, Medical University of Lodz, 92-213 Lodz, Poland; 2 Institute of Materials Science and Engineering, Lodz University of Technology, 90-924 Lodz, Poland; jacek.grabarczyk@p.lodz.pl (J.G.); witold.szymanski@p.lodz.pl (W.S.)

**Keywords:** endodontic instrument, fractured, removal, tube technique

## Abstract

One recommended technique for removing broken root canal instruments is to glue the fragment into a cannula adapted to it (i.e., the tube technique). The aim of the study was to determine the influence of the adhesive kind and length of the joint on the breaking force. During the investigation, 120 files (60 H-files and 60 K-files) and 120 injection needles were used. Fragments of broken files were glued into the cannula using one of three materials: cyanoacrylate adhesive, composite prosthetic cement, or glass ionomer cement. The lengths of the glued joints were 2 and 4 mm. After the polymerization of adhesives, a tensile test was carried out to find a breaking force. The results were statistically analyzed (*p* < 0.05). For 4 mm lengths of glued joints, the breaking force was higher than for 2 mm for both file types (K and H). In the case of K-type files, the breaking force was higher for cyanoacrylate and composite adhesives than glass ionomer cement. For H-type files, no significant difference in joint strength was found between binders at 4 mm, while at 2 mm, a much better connection was obtained for cyanoacrylate glue than prosthetic cements.

## 1. Introduction

Fracture of root canal instruments is a possible complication during endodontic treatment [1,2]. In a survey conducted in the United Kingdom of Great Britain and Ireland, nearly ninety percent of dentists (88.8%) admitted at least one endodontic instrument fracture [3,4]. Instrument fractures occur with both hand and rotary files (1–4). There are two main mechanisms of instrument breakage: fatigue failure and torsion failure [1]. Fatigue failure occurs as a result of cyclic bending loads resulting from the reciprocating or rotational movement of the file in the root canal. Torsion failure occurs when the tip of the working part of the file becomes wedged in the canal while the file continues to rotate.

Due to the imperfection of the instruments, the complex anatomy of the teeth (e.g., curved, narrow canals, and obliteration), and possible human error, fatigue and torsion failure can occur almost simultaneously. In some cases, a broken file can be left in the tooth. In many situations, however, an attempt should be made to remove the broken fragment of the endodontic instrument [5,6]. Currently, various methods are used to remove broken tools. One of them is the so-called tube technique. Various ready-made kits for removing broken tools using this method are available on the market, sometimes with various modifications.

One of the oldest and best-known systems is the Masseran kit, introduced about 50 years ago. It consists of a trepans with a diameter of 1.2 mm to 4.4 mm, an extractor, and forceps. Using the trepan, a tunnel is cut around the broken instrument for half its length. An extractor is placed over the protruding fragment of the instrument and carefully unscrewed counterclockwise. This method is relatively simple, but the whole kit is expensive. In addition, it is useless in narrow canals with a thin layer of dentin and in curved canals because its use may cause perforation of the root. Furthermore, the Masseran kit cannot always be used when broken files need to be removed from the posterior canals or when patients are unable to open their mouths wide. Due to the length of the individual instruments, it is not always possible to manipulate the fingers in more difficult situations [7]. The Meitrac set (Hager and Meisinger, Neuss, Germany) also uses the tube technique. The principle of operation is the same as in the Masseran kit. The Meitrac set also consists of trephines and extractors, but their diameter is smaller: 0.15–0.9 mm. The tube technique was also used by the creators of the Endo Rescue kit (Komet/Brasseler, Savannah, GA, USA) [8]. It consists of five instruments, and each of them has a different purpose. The set includes both instruments to create access to the broken fragment of the file as well as instruments to seize the fragment and remove it. The Endo Rescue Kit contains smaller instruments than the Masseran kit; however, the components of all the above-mentioned systems are rigid and large enough in diameter that their use is often limited to front teeth, wide and straight canals, and/or only the coronal part of the canal.

A system that uses the philosophy of the tube technique but with some modification is the Removal System (IRS) Superior EditionTM (Dentsply, Ballaigues, Switzerland) [9]. The set includes instruments called Retriever (IRS Assembly) and Core Drills. Retriever instruments range from 0.4–0.6 mm to 1.0–1.3 mm, and Core Drills from 0.61–0.41 mm to 1.7–1.4 mm. Core Drills are first used to expose a broken file section, and then a Retriever is introduced into the root canal.

It has a characteristic window, which plays a significant role in anchoring the broken fragment in the sleeve. The limitations of using the IRS system are similar to those of the Masseran kit [10]. As instruments break not only in straight and wide canals, and the dentist is often under pressure when performing the tube technique, improvements were proposed using appropriately modified hypodermic surgical needles or injection needles [11,12,13]. This modified tube technique, using the above-mentioned needles, is sometimes called the microtube method. Thanks to the relatively small size of the needles, this tube method can also be used in situations where the use of ready-made sets would be impossible. It should also be remembered that the needles come in many sizes, can be adapted to different diameters of broken instrument fragments and dentin thickness, and can be bent at different angles to suit the current needs. An additional advantage is the much lower cost of the needles themselves compared to factory sets. As in the case of ready-made sets, in the tube technique, it is necessary to expose the broken part of the instrument so that the tube (cannula) can be put on it. The length of the exposed fragment depends on the anatomical conditions of the tooth, the risk of possible complications, e.g., root perforation, and the ability to grip the broken tool [10,11,12,13]. To remove the broken instruments, a binder (adhesive) must be used between the inner wall of the cannula and the removed fragment of the file [14]. In clinical work, doctors very often encounter situations when the glued connection is torn and the cannula is removed from the canal with the broken tool still remaining in the tooth. This situation, which is quite frustrating for the dentist, prolongs the procedure and often makes it necessary to arrange another appointment for the patient. This generates additional costs and is time-consuming for both the doctor and the patient. To optimize the quality of the connection, various binders are used, most often prosthetic cement [13,14,15].

Some authors also recommend the use of cyanoacrylate glue [14]. The latter material has gained great popularity in industry, everyday life, and even medicine, including dentistry [16,17,18,19,20]. In addition to the undoubted benefits of using cyanoacrylate adhesives, one should also remember the limitations of their use in medicine. Especially if they are used directly on soft tissues, further research is needed to ensure their safe use [17]. Due to their excellent bonding properties, adhesives based on cyanoacrylates are currently used in dentistry, among others, in periodontal, surgical, and endodontic procedures [18,19]. Cyanoacrylate adhesives provide very good connections between various types of materials, including metals and their alloys. These are materials from which injection needles and endodontic tools are made. Therefore, they are often used during the removal of broken tools with the tube technique [11,14,15,20]. Some authors stipulate that cyanoacrylates are most effective when the space between the glued objects is less than 1 mm. It has been proposed that the type of broken instrument may influence the quality of the cannula connection. Due to differences in the construction of files, there may be differences in the value of the force needed to break the needle-file connection [14,15].

The aim of the study is to compare the strength of the bond between the endodontic file and the cannula using three binders and two types of K and H stainless steel files.

### 1.1. Null Hypothesis

The quality of the connection between the broken file fragment and the injection needle is independent of the length of the file fragment glued inside the needle, the type of binder, and the type of endodontic file (K files vs. H files).

### 1.2. Alternative Hypothesis

The quality of the connection between the broken fragment of the file and the injection needle varies depending on the length of the fragment of the file glued inside the needle, the type of binder, and the type of endodontic file (K files vs. H files).

## 2. Materials and Methods

**Figure 1:**
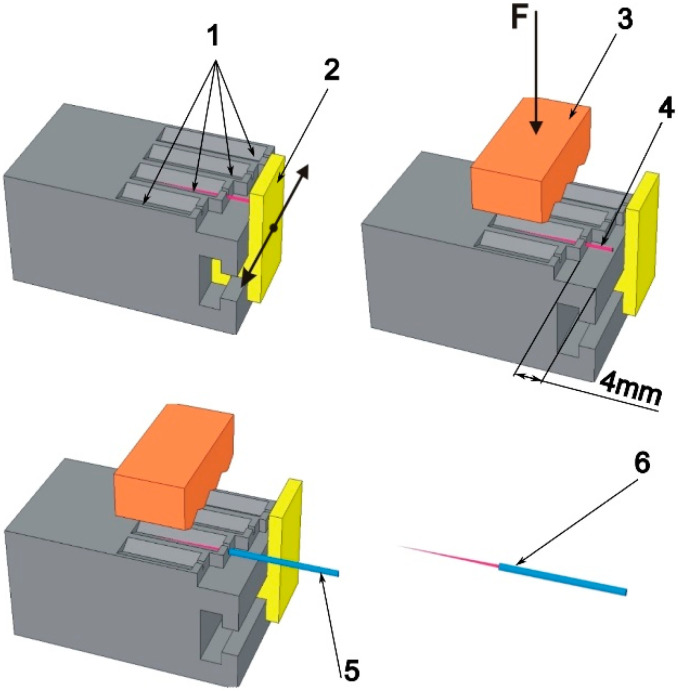
A device for gluing file fragments into a cannula for a specific length; 1—v-blocks; 2—sliding file stop; 3—clamp; 4—file placed in a v-block (free end of file 4 mm); 5—cannula placed on a fragment of the file; 6—ready file-cannula set for testing the strength of the glued joint.

One hundred and twenty files (60 H-type files and 60 K-type files) and 120 injection needles (size G21) were used for the study (Table 1). All files were cut behind the working part, thus creating file fragments of similar lengths (~16 mm). Furthermore, the tip of the injection needle was cut, leaving only the cannula with an outer diameter of 0.8 mm (inner diameter ~0.6 mm). The file fragments were then glued to the prepared cannulas (sleeves). For precise gluing, the device shown in Figure 1 was designed and printed on a 3D printer. The device shown in Figure 1 was used for precise gluing of a broken file with a cannula. The 3D model of the device was created in CAD software (Designspark Mechanical 6.0, RS Designspark, London, UK) and printed on a 3D printer Ultimaker 2+ (Ultimaker B.V., Geldermalsen, The Netherlands) using FDM (Fused Deposition Modeling) technology. Polylactide (PLA) polymer filament (Ultimaker B.V., Geldermalsen, The Netherlands) was used for 3D printing.

File fragments were placed in the v-block of the device and moved to the stop. Following that, the file was pressed down with a clamp, the stop was moved away, and the cannula was filled with a suitable binder and put on the file to a length of 2 mm or 4 mm. One of the following materials was used as a binder: standard cyanoacrylate adhesive (CA) (Kropelka, Bripox, Warsaw, Poland), glass ionomer cement (GC Fuji Plus, GC^®^ = GCFP), and chemically cured composite cement (Maxcem Elite, Kerr^®^ = MCE, Brea, CA, USA). Some of the sets of glued joints of H and K files with cannulas were embedded in epoxy resin. Furthermore, the samples were grounded and polished to obtain longitudinal cross-sections of glued joints. With the use of a light microscope (Nikon MA-200, Tokyo, Japan), the glued joints were assessed. The actual embedding length and true length of glued joints were measured. The embedding length was measured as the length of fragment files inserted into the canula. The true length of the glued joint was measured in such a way that the measurements of joint length start or end in the area of lack of adhesive on both sides of the endodontic file.

A sample size calculation was performed before the commencement of the research. Sample size calculations were made with the application of the Statistica 10.0 software (StatSoft, Hamburg, Germany), considering the following: *p* = 0.05, power test = 0.90, results presented by Wefelmaier et al. [14], and assumptions of our own experiment. The experiment was carried out on ten samples in the group.

After polymerization, the compound between the tubes and the endodontic instruments was used for pull-out tests (static tensile tests). The results were statistically analyzed (Kruskal-Wallis test, Mann-Whitney U test, *p* < 0.05).

## 3. Results and Discussion

In our study, the null hypothesis was rejected and the alternative hypothesis was retained.

**Figure 2:**
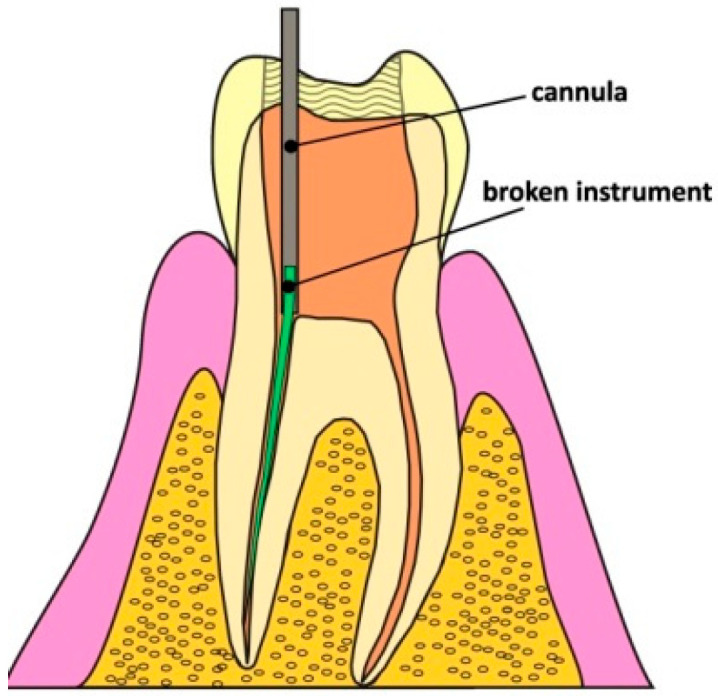
Access in a straight line to the broken fragment of the file and the cannula mounted on the broken instrument.

Cannulas obtained from injection or hypodermic needles are commonly used to remove broken root canal instruments using the tube technique [10,11,12,13,14,15]. In clinical conditions, before attempting to remove a “foreign body” using this method, a thorough analysis of the position of the broken fragment of the tool in the tooth cavity should be made to determine whether it will be possible to make a safe access in a possibly straight line (Figure 2) [10].

In some cases, there is some free space around the broken instrument that can be used to seat the cannula. If, in addition, access to the instrument is in a straight line, this is an ideal situation. In this case, the broken instrument can be removed without any additional damage to the root dentin. Unfortunately, such situations are very rare. Most often, a fragment of the broken file needs to be exposed, for example, using ultrasound, and only then can the procedure of removal using the tube technique be started. The correct access and connection are shown in Figure 2. The dentist must ensure that the inside diameter of the needle and the outside diameter of the broken file are well matched. Thanks to this, a good connection can be obtained [11,12]. Before the experiment, various cut injection needles were fitted to the outer diameter of the broken file, and it was found that the size G21 needle was optimal. The strength of the glued connection depends, among others, on the adhesive forces of the adhesive to the glued surfaces, the cohesive forces in the gluing, the thickness of the gluing, and the size of the glued surface. The average clearance in the glued section (4 mm), measured from the cutting tips and the core of the K-type file, is ~18 and ~86 µm, respectively. For H-type files, these clearances are ~19 and ~148 µm, respectively. This ensures the optimal thickness of the adhesive layer, which for most adhesive substances should be between 50 and 150 µm [21]. This proves that the diameter of the broken tool is optimally matched to commercially available cannulas obtained from injection needles.

**Figure 3:**
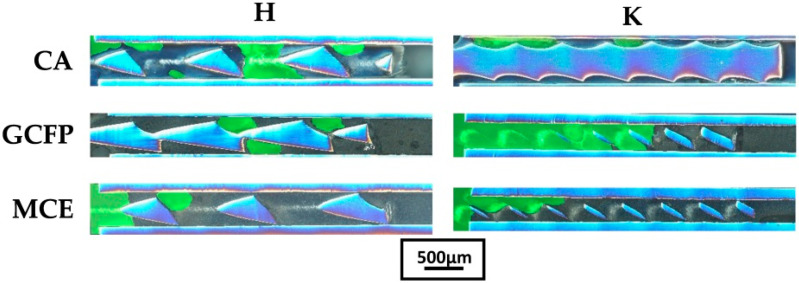
Longitudinal cross section of K- and H-type files glued with cyanoacrylate glue (CA), glass ionomer (GCFP), and composite cement (MCE). The scale bar is for all presented photos.

**Figure 4:**
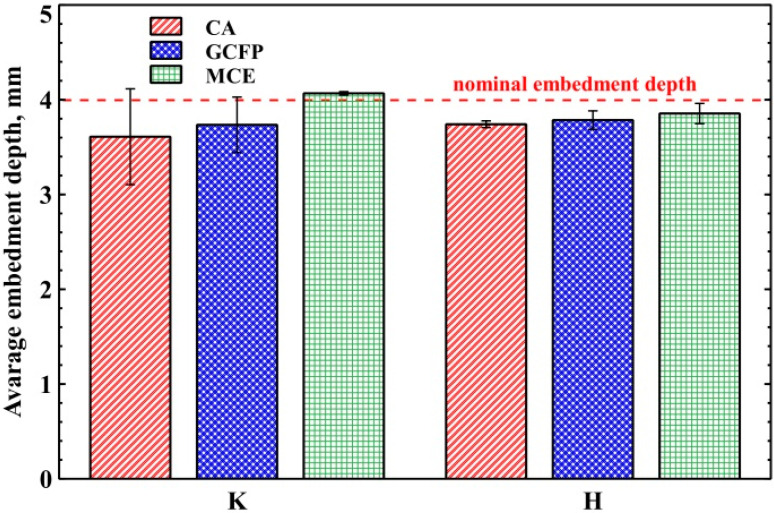
Depth of file insertion in the cannula for various adhesives and file types K and H.

The larger the surface of the bonded joint (file-cannula), the greater the probability of clinical success. Of course, in a clinical situation, it is always necessary to carefully consider whether a larger fragment of the file can be exposed [18,19]. In the present study, two file insertion lengths, viz. 2 and 4 mm, were used for the cannula. The files were glued to the desired length (2 or 4 mm) using a special device that allowed precise insertion into the cannula. In order to check the insertion depth, a longitudinal cross section of the file-cannula set embedded in epoxy resin was made to a nominal joint length of 4 mm (Figure 3), and the depth of insertion was measured by photos of cross-sections. The mean depth of insertion of the file into the cannula was 3.76 ± 0.24 mm (Figure 3). No significant difference was found between the groups (ANOVA; *p* = 0.6017), confirming that the matched method and file sticking device were effective and characterized by high accuracy (Figure 4). However, it is necessary to distinguish between the depth of insertion of the file in the cannula and the true surface of the connection between the file and the cannula, which is perfectly visible in Figure 3 in the cases of K-GCFP and H-MCE.

This state of affairs is probably greatly influenced by the viscosity of the adhesive used. The rheological properties of the adhesive are of great importance when it flows into narrow gaps and surfaces with irregularities. In the cases considered, the low viscosity of the adhesive will be conducive to filling the gaps created as a result of fitting the file with the cannula. Cyanoacrylate adhesives, among others, have low viscosity. Cyanoacrylates are one-component, room-temperature curing adhesives that are available in a variety of viscosities, from water-thin liquids to thixotropic gels [22]. The dynamic viscosity of cyanoacrylate adhesives can range from 0.005 to 5 Pa·s. The typical viscosity value of low- to medium-viscosity cyanoacrylate adhesives is 0.04 Pa·s. The viscosity of the glass and composite cements used in dentistry can range from 5 × 10^−5^ to 50,000 Pa·s [23,24]. Importantly, their viscosity changes very strongly over time. In contrast, the viscosity of water is 0.001 Pa·s. The viscosities of the used binders differed to a large extent, which was easy to notice when filling the cannulas. In the subjective opinion of the authors, the viscosity of the adhesives used was as follows: CA < MCE < GCFP. The influence of the viscosity of the used binders on their ability to fill the space between the file and the cannula can be clearly seen in Figure 3. Empty spaces (without glue) are marked green.

**Figure 5:**
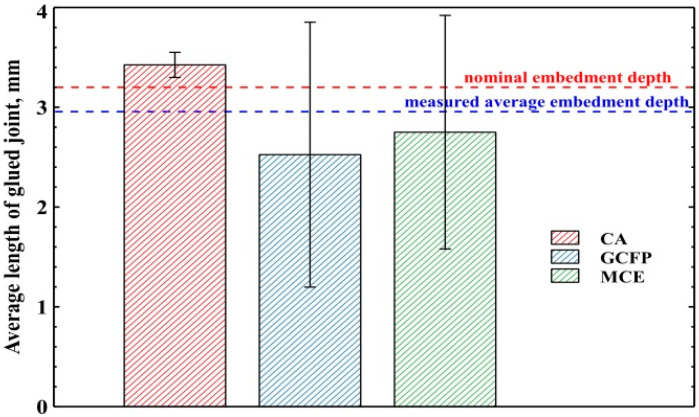
The average length of the glued file-cannula joint measured on the basis of the longitudinal cross section.

The length of the bond was measured in such a way that the length of the bond was taken to end at the point of lack of glue on both sides. Such a measurement, although it does not fully reflect the area of the glued surface, increases the probability of a lack of adhesive mass around the entire perimeter of the glued parts. The results are shown in the chart below (Figure 5).

The graph (Figure 5) clearly shows that the use of the lowest viscosity adhesive (CA) results in greater repeatability of the glued joints produced. Composite (MCE) and glass-ionomer cements (GCFP) are characterized by a similar average bond length and a similar dispersion of measured values.

This article also compares the size of the connection force between the needle and the broken files, depending on the binder used. K-type files demonstrated better binding quality for CA and MCE compared to GCFP at both embedding depths. In the case of H-files inserted to a depth of 4 mm, no statistically significant difference was observed in the strength of the file-cannula connection, regardless of the type of binder used. For the H-files at an embedding depth of 2 mm, a clearly stronger bond was achieved with CA compared to the prosthetic cements. No statistically significant differences in the strength of the bond made with GCFP and MCE were observed for H files with an embedment depth of 2 mm (Table 2).

So, the conducted experiments show that the cyanoacrylate adhesive is a very good alternative to the prosthetic cements used in the case of removing both broken K and H files for both embedding depths. If it is necessary to remove K-files from the canal, the best option is to use cyanoacrylate glue or Maxcem Elite (MCE) prosthetic cement. Maxcem Elite is a composite cement. Thanks to the chemical binding process, no polymerization lamp is needed. In addition, it is a self-etching material and does not require the use of any binding systems or additional preparation of the glued surfaces. This applies to both prosthetic cases and the use of this cement to remove broken files using the tube technique. An additional advantage of the above-mentioned material is the fact that it is produced in packages with a self-mixing tip, thus reducing the risk of procedural errors during material processing.

In the present study, the worst bond quality was observed after the use of GC Fujii glass ionomer cement (GCFP), especially with K-files. In the group of H-type files with an embedding depth of 4 mm, no significant differences in the strength of the glued joint were found. It is worth noting, however, that the absolute values (mean and median) were worse in the subgroup where the GCFP material was used. In the group where glass ionomer cement was used, no prior surface preparation was required. Files were also glued in without surface preparation using the two previously discussed binders. Although this contributed to the standardization of the tests (no additional modification of the file and needle before the application of the binder), it could have contributed to worse results in the group where GCFP cement was used. The manufacturer of GCFP recommends additional preparation of the tooth surface with a conditioner in order to improve the bonding quality. No conditioner was used in our experiment, and it is not known whether its use would have any effect on the quality of the connection between the needle and the file. The reason for not using the conditioner in this experiment is the fact that the use of the conditioner in clinical conditions could cause complications during treatment, e.g., the cannula and the file sticking to the tooth tissue. Bürklein et al. compared the force needed to break the file-cannula connection with and without additional surface modification prior to the application of a light-cured composite. The study compared GC Metalprimer, Prime, and Bond active, NaOCl (3%), citric acid (15%), and phosphoric acid (37%). It was found that Prime and Bond active significantly increased the adhesion of the light-polymerized composite to the surface of files made of nitinol (NiTi). It should be noted that the authors used a slightly different material (binder) and nickel-titanium files instead of steel, so their test results cannot be compared with our present findings. Wfelemeier et al. found that composite cements provided a better bond between the broken file and cannula compared to cyanoacrylate adhesive. It is worth noting, however, that different types of composite cements were used: Rebilda (DC; VOCO, Cuxhaven, Germany) and SDR (Dentsply, York, PA, USA). Rebilda is a dual-curing composite cement, and SDR is a light-curing only cement. Polymerization of the SDR composite took place thanks to optical fibers inserted into the cannula instead of the glued connection. No irradiation was used for Rebilda. The authors found the light-curing composite offered a better connection than the dual-curing composite cement [14].

One of the evaluated relationships was to compare the breaking forces of the glued connection of broken fragments of endodontic files with the cannula depending on the length of this connection. The results of our experiment clearly showed that the strength of the connection between the file and the needle is significantly greater if the depth of insertion of the file in the cannula is greater. The force needed to break the connection was on average about twice as high in the group where the embedment depth was nominally 4 mm, compared to the group where it was 2 mm (*p* < 0.05) (Table 2). The null hypothesis was thus rejected and the alternative hypothesis retained. Similar conclusions were presented by Wefelmeier and colleagues [14]. They found that the glued joint was stronger at greater embedding depths, regardless of the adhesive substance or file type [14]. In clinical conditions, the length of the glued fragment of a broken file depends, among other things, on the possibility of safely exposing the foreign body at the desired length [16,17]. The possibility of inserting a longer fragment of the file into the sleeve directly translates into a stronger connection of the needle with the file and increases the chance of removing the broken fragment [19].

Our paper also analyzes the effect of the type of file on the quality of the glued joint, assuming that the files are glued to the same length with the same glue. After applying the cyanacrylate adhesive, no statistically significant differences in the strength of the bond were noted, regardless of whether the H or K files were removed (no statistically significant differences were found between the two types of files at 2 and 4 mm). The results for prosthetic cements are different. After using the GCFP material, a better connection was found (greater force needed to break the needle-tool system) for H files than K files (the difference was statistically significant at both 2 and 4 mm). However, after using the MCE material, no such differences were found between K and H files at a length of 4 mm, while a significantly better connection was observed with K files at a length of 2 mm (Table 2).

Differences in the strength of the connection between K and H files are probably due to significant differences in their construction (Figure 3), which may result in a more favorable distribution of the adhesive in the spaces between the cutting edges and the cannula. The distance from the wall of the cannula to the core of the wire from which the file is made differs between the K and H files. They assume higher values for H files (about 148 µm) than K files (86 µm).

Our findings may help dentists better understand the tube technique and the relationships leading to the implementation of more effective clinical practices.

If it is necessary to remove an H-type file from the root canal and if ~4 mm of the broken instruments can be exposed, it does not matter what material will be used as a binder. If it is possible to embed the H-type file in the cannula to only ~2 mm, cyanoacrylate glue should be chosen instead of the prosthetic cements presented here. If the dentist does not have cyanoacrylate adhesive available, it is better to use Maxcem Elite cement to remove the K-files from the root canal. In the case of H-files, the connection made with Maxc em Elite cement proved to be as effective as with GC Fujii cement. However, both cement joints performed worse than the joint made with cyanoacrylate adhesive.

To date, very few experimental studies have evaluated the cements and binders used in the tube technique. However, one noteworthy study examined a very specific problem related to the removal of broken nickel-titanium files using a modified tube technique with a light-curing composite [25], the aim being to determine the influence of polymerization cycles and mechanical exposure procedures on the adhesion of instrument fragments. The results indicate that both the number of polymerization cycles and the mechanical exposure procedures had a significant impact on the adhesive force; the failure load increased significantly with the number of polymerization cycles (*p* < 0.0001). More than four polymerization cycles had no further benefit (*p* < 05).

Most articles are case reports concerning the removal of broken files with a needle/cannula [26,27,28,29,30,31,32]. They describe the use of various binders such as polycarboxylic cement, composite cement, self-adhesive, and self-healing types [26,27,28]. It is worth noting that many authors also use cyanocarrylate glue, and some, instead of cement or glue, used an additional file to anchor the broken fragment of the instrument inside the injection needle [26,29,30,31,32].

However, regardless of the type of binder, it should be remembered that it is difficult to remove broken instruments using an injection needle in clinical conditions. It requires experience, time, and specialized instruments. The previous studies indicate a fairly high percentage of successful broken root canal tool removal using the tube technique, either in the form of ready-made kits or the use of an injection or hypodermic needle [10,11,12,13,14,15,26,27,28,29,30,31,32,33,34]. However, it should be remembered that the authors of these articles are usually experienced dentists who specialize in root canal treatment and the treatment of difficult endodontic cases.

As mentioned earlier, for the removal of the broken fragment of the file to be possible at all, it must first be visualized in the canal and “uncovered”, typically with the use of ultrasonic tips; in addition, the entire procedure must be performed under the magnification of the operating microscope. Despite the relatively cheap materials, such as injection needles or used binders, in practice, the entire procedure generates higher costs related to the purchase and use of additional necessary instruments [35]. It is also necessary to use the microscope and ultrasonic devices appropriately and skillfully. The procedure is also time-consuming and requires patience [26,36,37]. Sometimes it is necessary to make an appointment for the patient, even for several visits, for the whole procedure to be successful. It may only be possible to visualize a broken file or remove a fragment during the first visit, with the broken part of the instrument remaining in the tooth. Patients should also be very carefully selected for surgery to remove a broken instrument. The greater the curvature of the canal and the “more deeply located” (closer to the root apex) the broken file fragment, the greater the risk of complications, especially perforation. In some situations, the risk associated with the removal of files is so great that a broken instrument should be left in the tooth, and after thorough disinfection and filling of the canals, the tooth should be examined. Only in the event of complications should a decision be made, for example, on endodontic-surgical or surgical treatment [1,2,3,4,10,38,39,40,41,42].

In addition to perforation, the procedure may result in the root canal becoming “clogged” by an uncontrolled leakage of the binder outside the cannula. Even in the case of small leakages, all adhesive residues must be removed from the tooth [10,26,29]. Here again, high precision and appropriate equipment (ultrasound, microscope) are necessary.

When working with ultrasound, care must be taken with the power setting, and copious rinsing must be used to cool the tooth structure and adjacent anatomical structures. Improper use of ultrasonic instruments may result in the breakage of the ultrasonic file itself in the canal as well as potential periodontitis and bone inflammation due to excessive temperature on the external surface of the root. According to the literature, the power of ultrasonic devices should not be higher than about 30%. To reduce the risk of excessive temperature increase on the outer surface of the root, it is recommended to rinse the root canal abundantly and frequently, e.g., with sodium hypochlorite solution [10,39].

Regardless of the number and frequency of rinses used, the color of the root dentin should be observed during the procedure; if a yellow-brown discoloration appears, the canal should be immediately rinsed with a cold agent, and the power of the device should be reduced [10,39,40]. Similarly to the removal of broken instruments and their “loosening” or exposure, the extraction of adhesive residues may lead to perforation or excessive weakening of the root wall, resulting in a tooth fracture requiring extraction [40]. Ideally, the material (glue) used during the removal of the broken file is visible on the X-ray, and intraoperative X-rays may be used in such cases. It is possible to trace the remaining areas to be cleaned on the images. To reduce the risk of iatrogenic complications, good-quality radiological images and the magnification of the microscope should be used [43,44,45].

In our work, because the experiment was carried out in typical laboratory conditions, in vitro, without tooth extraction, no surgical microscope was needed to glue the broken file fragment. Similar conditions were noted in earlier publications [14,15]. This approach allows for high standardization of research and avoids the potential influence of tooth anatomy on the results.

However, it was not possible to study any unmeasured displacement of the binder outside the cannula, which would limit or completely block the patency of the canal. The aim of the work was to determine the strength of the connection between the cannula (needle) and the broken file depending on the type of binder and the length of the insertion. It does not address any possible reaction between the cement and tooth tissues. In addition, the work does not deal with nickel-titanium alloy files, which are intended to be the subject of further research.

The findings can also be influenced by the strength of the connection between the file-binder and the needle, which may be determined by the chemical composition of the root canal instrument alloy as well as the large cross-sectional variation between machine files. As such, the results of the current study should be applied only to situations where steel hand files are removed using the tube technique.

## 4. Conclusions

1. Providing the largest possible bonding surface between the broken instrument and the cannula will enable the transfer of greater force, increasing the probability of removing the broken instrument from the canal.
2. Cyanoacrylate adhesive is a very good alternative to prosthetic cements. Our findings may help dentists better understand the tube technique and the relationships leading to the implementation of more effective clinical practices.

## Figures and Tables

**Table 1:** Division of file-cannula sets prepared for testing.

Group	Subgroup	Samples n	File Type	Joint Length, mm	Adhesive
1	a	10	K	2	CA
	b	10			GCFP
	c	10			MCE
2	a	10		4	CA
	b	10			GCFP
	c	10			MCE
3	a	10	H	2	CA
	b	10			GCFP
	c	10			MCE
4	a	10		4	CA
	b	10			GCFP
	c	10			MCE

**Table 2:** Obtained values of breaking force for all glued joints.

Endodontic File Type	Embedment Length	Adhesive	Average Breaking Force	Standard Deviation	Min	Max
	mm		N			
K	2	Cyanoacrylate (CA)	91.51 ^a1,a2^	40.27	48.241	155.135
		Max Cem Elite (MCE)	96.85 ^a1–2,c2^	33.20	49.6	150.657
		GC Fuji Plus (GCFP)	20.64 ^b1–2,f2^	7.87	11.341	36.928
	4	Cyanoacrylate (CA)	134.25 ^c1–2,b2^	14.18	107.29	152.315
		Max Cem Elite (MCE)	139.75 ^c1–2,e2^	10.08	133.116	167.52
		GC Fuji Plus (GCFP)	74.87 ^d1–2,h2^	20.04	43.71	108.61
H	2	Cyanoacrylate (CA)	88.31 ^e1–2,a2^	27.21	50.86	136.44
		Max Cem Elite (MCE)	64.59 ^f1–2,d2^	17.80	38.87	89.05
		GC Fuji Plus (GCFP)	56.2963 ^f1–2,g2^	20.12	30.098	95.14
	4	Cyanoacrylate (CA)	127.22 ^g1–2,b2^	11.39	107.28	144.35
		Max Cem Elite (MCE)	131.34 ^g1–2,e2^	21.13	91.04	158.39
		GC Fuji Plus (GCFP)	132.3563 ^g1–2^	7.82	120.12	142.44

The superscript letters show the significant differences. Different letters mean “statistically different” with the given significance level; the same letters mean “not statistically different” at a significance level of *p* < 0.05. ^a1–g1^—refers to the comparison of the force needed to break the connection of the system: needle-endodontic file (in pull-out test), depending on the length of insertion of the file and the type of glue. ^a2–g2^—refers to the comparison of the force needed to break the connection of the system: needle-endodontic file (in pull-out test), depending on the type of file (K file vs. H file).

## Data Availability

Not applicable.
